# Mandibular molar teeth deviations for fabrication of implant-specific strip crowns among Indians

**DOI:** 10.6026/97320630019436

**Published:** 2023-04-30

**Authors:** Kshitiz Chhabra, Sahana Selvaganesh, Thiyaneswaran N

**Affiliations:** 1 Department of Implantology, Saveetha Dental College and Hospitals, Saveetha Institute of Medical and Technical Sciences (SIMATS), Saveetha University, Chennai 600077, India

**Keywords:** Mandibular molar width, temporization of implant, strip crowns

## Abstract

An understanding of tooth morphology provides better insight and is an important foundation for providing successful endodontic and restorative treatment. It also provides a deeper evaluation and baseline for future prosthetic rehabilitation. The study aimed to measure and characterizes mandibular molars using a normal divider and measurement scale using mandibular casts. Over 300 mandibular first molars were measured in dimensions including the buccolingual width, mesiodistal width, mesiobuccal height, mid-buccal height, and distobuccal height; same were repeated with intra-oral scanners digitally. The mean and standard deviation of the measurements and the correlation to age and gender were calculated using SPSS software to fabricate implant-specific strip crowns.

## Background:

The different ethnicity and diverse genetic mixture in the population of India provides a wide range of scope for evaluation of the difference, genetics has on the morphological heights and width of teeth. Implantology is a field of precision, the amount of skill and evaluation required in the field by the practitioner is a base for successful prosthetic rehabilitation. The success of an implant is determined by the precision in the placement, evaluation of the space available for prosthetic rehabilitation, the amount of calibration required for the practitioner and the awareness of the dimensions of teeth they are replacing. [1,2] A balanced prosthetic rehabilitation provides the distribution of occlusal forces equally in vertical, horizontal and transverse forces. The amount of torsional strain prosthesis might undergo because of slightly improper design affects the life and stability of the implant in the long run. Proper knowledge of the morphology of the tooth and genetic variations that it brings can be a huge shot in the arm of the practitioner. Having the mesiodistal and buccolingual dimensions and variations of those with the ethnicity and different demographics can provide a well-set base for a fully functional and esthetically stable prosthesis. [3,4] Implants that are placed need temporization procedure to provide a base for the soft tissue to mold and hence aid in improvement of aesthetics, enhance the emergence profile. It provides an ideal collar around the tooth for a permanent crown to be given. Temporary crowns help in the formation of gingival zenith and mold the gingival collar. Temporization helps in forming proper contours of the interdental papilla and also helps to provide a wide base for the crowns which cannot be achieved by a stock healing cap. [5,6] Defining the ideal tooth dimensions is difficult because of the variations of individual teeth due to wear and resorption and the difference in morphology and genetic variability in every individual. But determining a range provides the benefit of ideal proportions of height and width of the teeth which can then be changed according to the variability of the dimensions of teeth in the patient [8]. A thorough understanding of clinical crown length and dimension provides the practitioner an idea of the specific anatomic modifications required for the rehabilitation of the tooth. The study of dental models has provided invaluable insights for the in-depth evaluation of tooth morphology, clinical crown length, estimation of space for prosthetic rehabilitation, and also for evaluation of arch forms, sizes, crowding, and spacing between the teeth. It also has provided a valuable base for orthodontic studies. [9] The data from the study has been further used for the fabrication of temporary shells to facilitate the preparation of temporary crowns on dental implants. These shells help produce temporary crowns which considerably reduce the time taken to fabricate a temporary crown chair side and have esthetic contours. This novel technique helps in the crown fabrication that provides a better emergence profile. This short study aims to develop three specific sizes of mandibular first molar teeth strip crowns particularly associated with implants. [7,10]

##  Material and methods:

**Figure 1:**
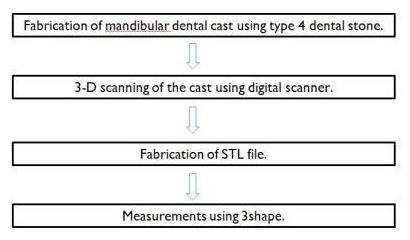
Showing procedure for digital measurement of dimensions.

This study (Figure 1) was conducted in the Department of Implantology, Saveetha dental college and hospital between January - December 2022 on patients who were undergoing dental implant surgical procedure. [11,12] Mandibular casts of 300 patients were allotted and vernier calipers was used to measure the dimensions of the first molar. The patients who had intact lower posteriors were selected as samples with an age range of 18-70 years. The sample population included 203 males and 97 females. A thorough investigation helps in determining the dimensions for restorative and future prosthetic rehabilitations. [13,14]

## Mandibular Impression and cast making:

For the fabrication of the casts, perforated stock trays were selected carefully for proper coverage of all the teeth, borders of the tray were evaluated for ideal sulcus depth. The impressions of the mandibular arches were made using putty and light body addition silicone following the manufacturer's instructions; impressions were then disinfected using a 2% glutaraldehyde solution for 5 minutes and were poured using type 4 dental stone. The cast was removed from the impressions after 30 mins. The base of the cast was formed and provided a serial number for identification. [15,16]

## Digital Scanning of the casts:

The patients were subjected to intra-oral scanning using 3shape software. The scanned cast was then transferred to 3shape software and measurements were done using 3shape software. The buccolingual and mesiobuccal width was measured and cross-verified with the manual measurements of the same casts. The digital scan measurement and evaluation were done and correlated with the manual measurements. Similar to the manual method, mesiobuccal, mid-buccal, and distobuccal heights and buccolingual and mesiodistal width were measured and correlated against manual measurements, and the results were obtained. [17,18]

## Dental Cast teeth Width and Height Measurement:

The measurements were taken by a single operator. Each value was measured three times and a mean of the values was noted. All measurements were made using vernier calipers and calculated using a divider and scale for accuracy of the results and the same measurements were done using digital scans of the same cast and using 3shape software for individual measurements.[19,20]

## STL file fabrication and printing:

Using these measurements, mean values were calculated. Three different sizes of mandibular molars were fabricated for different sizes of teeth for the south Indian population. Following that, the designing of strip crowns was done and STL (stereo lithography) files were made. The fabricated STL files were then 3-D printed using flexible resin and then used for the fabrication of temporary crowns. [21,22]

## Results:

**Figure 2:**
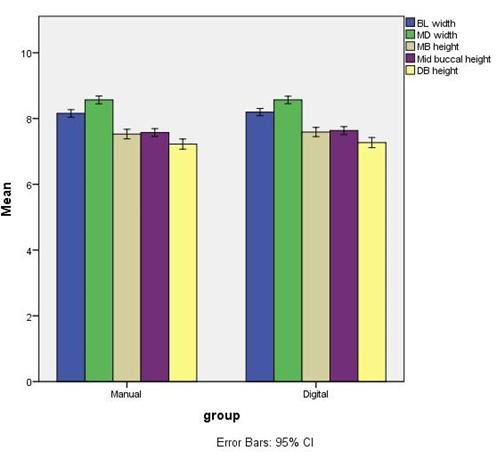
Comparison of measurements where Group 1 is a manual method of measurements and Group 2 is digital scanning and measurement.

SPSS software was used to calculate the mean age, buccolingual, mesiodistal width, and mesio-buccal, mid-buccal, and disto-buccal heights. The distribution of data (Figure 2) was analyzed with normality plots and testing. Analysis of descriptive data like gender, age, teeth width, and variables were performed to calculate mean and standard deviation and correlation between the age and gender of the patient with the morphology of the teeth. Interpretation was done using P value of ≤ 0.05 as statistically significant. Based on the calculations the first molar was classified into three sizes- small, medium, and large. These sizes were then correlated with the respective gender and age of the patient. The study showed that the mandibular first molar had a mean mesiodistal width of 8.15 ± 0.98mm, buccolingual width of 8.56 ± 1.04mm mesiobuccal height of 7.5 ± 1.27mm mid-buccal height of of 7.57 ± 1.04mm and the distobuccal height of 7.22 ± 1.36mm.

After evaluation of data using the SPSS software of both digital and manual methods, the software revealed no significant difference between the measurements done using manual and digital methods. Henceforth, the study was done using the manual method as the base for further calculations.

The mean of all dimensions calculated with standard deviation was correlated with age group and evaluated with the highest frequency distribution. Table 1 shows the corelation of age and corresponding dimensions. The age groups were divided as shown in Table 1. The dimensions showing the highest amount of recurrence with the corresponding age group provided the base for correlating age and various dimensions of the mandibular molar. It was also noted that there were no major differences between the sizes of the molar teeth, based on the inter-tooth and inter-occlusal space available, post implant placement the small, medium and large strip crowns were used. The fabricated strip crowns had an outer diamer (OD) and inner diameter (ID) varying height (H), the dimensions used to fabricate the strip crown are given respectively., small (OD-8.25mm, ID-7.5mm, H-7mm) , medium (OD-9.25mm, ID-8.5mm, H-8mm) and larger (OD-10.25mm, ID-9.5mm, H-9mm). Similarly, a correlation of the gender of the patient with the corresponding dimensions was done to evaluate the correlation between gender and dimensions of molars. Table 2 shows the correlation of gender and different widths of mandibular molars. The calculations done using SPSS software showed that female population had smaller dimensions of teeth compared to the males in South Indian population.

## Discussion:

Attempts have been made to reduce possible sources of error and bias. The limitation of the study is the small sample size and restriction to the mandibular first molar tooth particularly. The amount of human error has been reduced by making sure the collection of data done by one operator. The margin of error of the instrument might be inherently present but it doesn't seem to affect the data at a larger scale. The results of the present study are generalizable to the South Indian population and South Asian populations. The sample collection for this study has been done from the general population presenting to the OPD of Saveetha Dental College, Chennai, Tamil Nadu. A study repeated in the same population with a different sample should yield similar results. Subjects with different ethnicities may yield different results.

In 2022, Naseer Ahmed *et al*. showed the analysis of maxillary anterior teeth crown width-height ratios: a photographic, three dimensional, and standardized plaster model. The study analyzed width and height ratios of maxillary anterior teeth at different crown levels using photography and models were constructed for width and height analysis. The study was limited by its design and included only teeth in the esthetic zone. Also, the ratios described in the study provided an idea about the ratio of height and width. It did not take into consideration the variability of height and width and different places of the tooth. Also, fabrication of ratios meant the tooth was considered similar to a cuboid with uniform dimensions which is not the case morphologically. This study henceforth includes dimensions of the teeth considering five different locations including both height and width of the crown. Also, the current study serves as a base for characterization of the tooth into three different sizes which helped in the fabrication of strip crowns for the development of temporary crowns serving as an ideal to lay down the base for the values received from the study. [23]

In 2020, Xiao Jie Yin *et al*. in his article titled correlation between clinical parameters of crown and gingival morphology of anterior teeth and periodontal biotypes, included quantitative analysis of clinical parameters of crown and gingival morphology of maxillary anterior teeth. They also analyzed the correlation of these parameters with periodontal biotypes to provide objective standards for periodontal biotype diagnosis. The study has focused on the determination of crown height and width and correlating it with the gingival biotype of the patient. It provides an insight into how the crown and gingival biotype is interlinked and hence the adaptation of the concept to evaluate the soft tissue formation around an implant when the dimensional accuracy of the temporary crowns is higher and can be evaluated which was the core of the current study. The insights of how the crown dimensions revealed the correlation between cervical gingival margins of mandibular anterior teeth on both sides and how they are symmetrical and thin biotype accounts for small proportions. Gingival angle of 95.95 and papilla width of 10.01mm are the optimal cutoff values for characterization of individuals as thick biotypes. This data provides valuable insight for practitioners to classify the gingival biotype of the patient and the expected results post-final prosthesis insertion in the oral cavity of the patient. But the study came with similar limitations that it focused only on anterior teeth and restricted sample size. [24]

In June 2015, Phonepaseuth *et al*. in his article titled comparison of maxillary anterior teeth crown ratio between genders in the Laotian population. It aimed to determine the distribution of facial types and to compare the crown width/length ratio of six maxillary anterior teeth between males and females in the Laotian population. The study characterized facial types and crown width and length ratio were taken. The study also focused only on anterior teeth and the ratios did not provide specific variations in height and width through the mesiodistal length of the tooth. [25]

The studies mentioned above had restrictions to anterior teeth and the aesthetics concerns were limited to anterior tooth region. This study provides an insight to the esthetic needs of posterior teeth and a method to reduce the time involved in temporization of the teeth following implant. This study also takes into consideration the different techniques of impression making and that the difference in manual and digital methods was not significant when providing the measurements of mandibular first molar. It also helps in characterizing mandibular molars into small, medium and large for fabrication of temporary shells.

## Data availability:

The raw data used to support the findings of this study are included in the article and will be made available on request.

## Conclusion:

This study revealed the data collected using manual and digital method as not having any significant difference and helps characterization of mandibular molars into three different sizes. Also age and gender plays a significant role in the size of tooth and morphology of the tooth.

## Figures and Tables

**Table 1:** Maximum Frequency of age and corresponding dimensions (manual method)

**Age group (years)**	**Mesio-distal width (mm)**	**Bucco-lingual width (mm)**	**Mesio-buccal height (mm)**	**Mid-buccal height (mm)**	**Disto-buccal height (mm)**	**Total sample**
1. (17-30)	8.57 ± 1.065	8.5 ± 0.936	8.5 ± 1.229	8.0 ± 0.975	8.0 ± 1.319	166
2. (31-40)	8.63 ± 0.969	8.17 ± 0.966	7.44 ± 1.368	7.43 ± 1.142	7.06± 1.501	78
3. (41-50)	8.50 ± 0.969	8.44 ± 1.102	7.94 ± 1.184	7.78 ± 1.003	7.72 ± 1.034	9
4. (51-60)	8.59 ± 1.218	8.07 ± 1.083	7.48 ± 1.379	7.57 ± 1.075	7.17 ± 1.410	29
5. (61-70)	8.43 ± 0.732	8.14 ± 0.988	8.07 ± 1.170	8.00 ± 0.500	8.00 ± 0.500	11
6. (71-80)	8.57 ± 1.048	8.16 ± 0.991	7.53 ± 1.281	7.57 ± 1.045	7.22 ± 1.366	7

**Table 2:** Maximum Frequency of gender and corresponding dimensions (manual method)

**Gender**	**MD width (mm)**	**BL width (mm)**	**Mesiobuccal height (mm)**	**Mid buccal height (mm)**	**Distobuccal height (mm)**	**Total sample**
Female	8.55 ± 1.084	8.11 ± 0.933	7.50 ± 1.271	7.62 ± 1.039	7.24 ± 1.333	93
Male	8.59 ± 0.889	8.32 ± 1.051	7.68 ± 1.223	7.58 ± 1.090	7.58 ± 1.090	207
